# The role of linguistic and cognitive skills in reading Chinese as a second language: A path analysis modeling approach

**DOI:** 10.3389/fpsyg.2023.1131913

**Published:** 2023-04-04

**Authors:** Lei Yang, Ying Xiong, Qi Chen

**Affiliations:** ^1^Department of Foreign Language, Xi’an Jiao Tong University, Xi'an, China; ^2^School of Foreign Languages, Shanghai Jiao Tong University, Shanghai, China

**Keywords:** L2 Chinese reading, linguistic skills, cognitive skills, morphological awareness, path analysis

## Abstract

This study examined the role of basic linguistic skills (vocabulary, syntax, orthography, and morphological awareness), basic cognitive skills (working memory), and higher-order cognitive skills (inference making and reading monitoring) in reading Chinese as a second language (L2). A total of 252 international students from Pakistan, Indonesia, Malaysia, and Laos were recruited, and a range of measures including a Chinese reading comprehension test (HSK level 3), four linguistic knowledge tests on Chinese lexical, syntactic, and orthographic knowledge as well as morphological awareness, a reading span test, an inference making task, and an inconsistency detection test. The results of hierarchical multiple regressions showed that the measured linguistic skills and cognitive skills explained 80% of the variances in L2 Chinese reading, among which morphological awareness made the largest contribution. The path analysis revealed that linguistic skills and working memory contributed indirectly to reading comprehension *via* inference making and comprehension monitoring, while the two higher-order cognitive skills made direct contributions. Overall, this study demonstrates that inference making and comprehension monitoring contributed directly to reading comprehension, while linguistic skills and working memory functioned indirectly *via* the higher-order cognitive skills It also highlights the importance of morphological awareness in a hierarchical model of L2 Chinese reading.

## 1. Introduction

Reading is a fundamental part of second language (L2) learning. International students learning in China also take Chinese reading as a way of cultural exploration. However, the remarkable differences between the Chinese language, ideography, and their native language may result in great difficulties in comprehension. In addition, complex cognitive processing underpins reading comprehension. It requires readers to understand not only the meanings of individual words but also ideas across sentences and larger stretches of texts. What factors may influence Chinese reading comprehension among international students in China? This study intends to explore a range of factors involved in Chinese reading comprehension and reveal the complex relations between language and cognitive skills. The findings of this study may provide insights into Chinese teaching and learning.

## 2. Literature review

### 2.1. Theories of reading comprehension

Studies of reading have been conducted at both word and sentence levels. At the word level, reading concerns how readers automatically map printed forms of words to the semantic meaning of words and initiate orthographic decoding, phonological encoding, and semantic integration (Frost et al., 2008). At the sentence level, the crux of the matter is how words are related to their mental representations. According to the reader’s situation model (Van Dijk and Kintsch, 1983), readers need to integrate related words into a comprehensible conceptual structure to achieve sentence level comprehension as their goal is beyond the mapping between forms and meanings. Therefore, readers need to construct a series of closely related conceptual units and establish a hierarchy of themes as their mental representations rather than a loose set of concepts (Fesel et al., 2015; Kim and Clariana, 2015, 2017).

Taking reading as the construction of situation models rather than form-to-meaning mapping does not diminish the importance of word knowledge in reading. On the contrary, it is vital for readers to integrate word meanings into relevant conceptual units. Reading System Framework (RSF, Perfetti and Stafura, 2014) encompasses both linguistic knowledge and cognitive skills, providing a framework that explains word-to-text comprehension processes. In RSF theory, three key hypotheses are proposed:

1. Three types of knowledge sources are employed in reading ranging from basic linguistic knowledge to general knowledge.
2. The processes of reading include decoding, word identification, meaning retrieval, constituent building (sentence parsing), inferencing, and comprehension monitoring.
3. These cognitive processes occur within an interactive processing system that has limited resources in terms of attention, memory, and control.

According to RSF theory, reading comprehension relies on basic cognitive function, fundamental linguistic knowledge, and higher-order cognitive functions. There is a hierarchical relationship between these language and cognitive skills. In terms of hierarchical relations, inference making is considered as a higher-order cognitive skill directly related to reading comprehension, as higher-order cognitive skills are capable of integrating multiple information across discourse to form a coherent whole (Strasser and Del Río, 2013; Kim, 2016). Comprehension monitoring is another higher-order cognitive skill that involves reflecting on and regulating an individual’s understanding of the incoming information. Readers use it to evaluate whether their understanding of the newly received information is coherent with those from the previous portion of the discourse and to resolve any discrepancies. In addition to the skills mentioned above, working memory, referring to the basic cognitive resource for processing general information, was also considered an important contributor to reading comprehension (Kim, 2016). Working memory defined as the capacity to store and manipulates information is the key to how efficiently a reader can hold, process, and recall information in mind (Seigneuric et al., 2000).

Reading comprehension is supported by cognitive skills, which, in turn, are supported by foundational language skills. Foundational language skills construct initial and literal propositions based on the words and phrases of the discourse. Foundational language skills are necessary for the construction of initial propositions by assigning meaning to individual words and sentences. But in many cases, the initial propositions are not complete and sometimes contradictory. Higher-order cognitive skills are necessary to integrate information across sentences, detect contradictions, and construct coherent mental representations (Kim, 2016, 2017; Oakhill, 2020).

In sum, reading comprehension is conceptualized as having three levels: the basic cognitive skill, foundational language skills, and higher-order cognitive skills. These language and cognitive skills have hierarchical relationships, which can be characterized as having direct and/or indirect routes towards reading comprehension. According to the RSF theory, cognitive skills (i.e., inference making and comprehension monitoring) are higher-order skills, directly related to reading comprehension. As foundational language skills (e.g., syntactic, lexical, morphological, and orthographic knowledge) are necessary to attach meaning to individual words and sentences, they should be placed at a lower level, contributing directly or indirectly to reading comprehension. The basic cognitive skill (i.e., working memory) that manipulates and stores the information in mind is placed at the same level as the foundational language skills.

### 2.2. Factors in reading comprehension

#### 2.2.1. The impact of foundational linguistic skills

Successful reading comprehension includes two phases: a foundation phase and a construction phase. Linguistic knowledge as a foundational skill for reading encompasses vocabulary, morphological awareness, syntactic knowledge, and orthographic knowledge to facilitate further information integration. In this section, we will discuss these different types of linguistic knowledge that are essential to reading comprehension.

Developing a strong vocabulary is at the core of reading comprehension. In order to make propositions, readers must be able to attach meaning to most words in a text, assisting the integration and construction of text meaning. Vocabulary is strongly correlated with reading comprehension (Cain et al., 2004; Seigneuric and Ehrlich, 2005). In previous studies, vocabulary was a strong predictor of reading comprehension among native Chinese speakers (Leong and Ho, 2008) as well as L2 Chinese learners (Zhang and Koda, 2018; Zhang et al., 2021).

Despite that vocabulary is foundational, in the absence of syntactic knowledge, the text cannot be understood. Syntactic knowledge provides readers with necessary rules to understand sentence structures, identify syntactic categories of words, and process text information at discourse level. A good understanding of syntactic structures facilitates the recognition of word meaning as well as sentence and discourse comprehension. Syntactic knowledge is crucial for Chinese reading due to the unique features of Chinese such as the absence of an inflectional system, more flexible word order, and more extensive use of connectives (Li and Thompson, 1981). Chinese readers rely on their syntactical knowledge to seek information about tense, number, and their semantic relations (Lin, 2006). Besides, word order is more flexible in Chinese. There are two main types of structure in Chinese: subject-verb and topic-prominent. The basic word order in Chinese is SVO. For example, in 我爱吃面条 (I love to eat noodles), 我 (I) is the subject, 爱吃 (love to eat) are two verbs serving as the predicate, and 面条(noodles) is a noun serving as the object. In the above sentence, the object 面条(noodles) can be moved to the beginning of the sentence to form a topic-comment sentence (面条，我爱吃), which means “As for noodle, I love to eat (it).” The topic can be extended to several subsequent sentences once it has been established (Chik et al., 2012). Therefore, Chinese readers often come across sentences without subjects. Another important characteristic of Chinese syntax is the extensive use of connectives indicating cause, time, and contrast. It is thus important to keep track of the logic and semantic relations between words and phrases within a sentence and also across sentences with the help of syntactic knowledge of word order and connectives. Therefore, Chinese readers rely on their syntactic knowledge to accomplish reading comprehension and information processing. Syntactic knowledge was found to be an important contributor to the comprehension of sentences and texts (Chik et al., 2012; Tong et al., 2014; Lo et al., 2016).

However, whether syntactic knowledge may exert an impact on Chinese reading *via* cognitive skills has not been examined in depth. Hung’s (2021) recent study represents such an attempt to find the intertwined relationships between syntactic skills and cognitive skills. She found an indirect effect of executive function (as measured by working memory tasks and inhibition tasks) on Chinese Grade 2 and 3 students’ reading comprehension through syntactic awareness (as measured by a word order correction task). However, no indirect effects of executive function were found on reading through inference-making, refuting her original hypothesis that executive function can support inference making to achieve passage-level comprehension. The question that remains to be answered is whether the intertwined relationships between language and cognitive skills remain the same among L2 Chinese readers.

Orthography also plays an important role in Chinese reading. Chinese has a morphosyllabic orthography that consists of characters, radicals, and strokes, and each character represents both a morpheme and a syllable (Shu et al., 2000). A character is composed of radicals, which in turn is composed of strokes. In Chinese characters, about 80% or so are phonetic-semantic (Shu et al., 2003), containing both semantic and pronunciation information. Semantic radicals reveal a character’s meaning, while phonetic radicals reveal a character’s sound. Chinese radicals at particular positions (top, bottom, left or right) determine or pertain to the sound or meaning of the character. The orthographic knowledge of Chinese can greatly promote beginner-level L2 readers of Chinese since a significant part of modern Chinese characters is regarded as semantic–phonetic compounds that contain both semantic and phonetic components (Shu et al., 2003). In Chinese, faster mapping occurs from orthographic information to semantic information than from orthographic information to phonological information (Yang et al., 2006). Empirical evidence has shown that for L1 Chinese children, Chinese reading comprehension was greatly influenced by their orthographic knowledge (Cheung et al., 2007; Yeung et al., 2016). A further question to be resolved is whether this is also the case for L2 Chinese readers.

Apart from the factors mentioned above, morphological awareness also plays a significant role in reading comprehension because it can help readers generate important clues about meaning in alphabetic languages like English and Spanish (Carlisle, 2000) as well as in the non-alphabetic language like Chinese (Packard, 2000). Morphological awareness is defined as the ability to reflect and draw on the componential units of words that carry meanings such as derivational and inflectional affixes in English (Carlisle, 2000; Kieffer et al., 2013). It has been well-established that morphological awareness is a crucial skill for achieving high-quality reading comprehension in alphabetic language such as English (Carlisle, 2000; Nagy et al., 2006; Tong et al., 2011; Kieffer et al., 2013). The samples of these studies mainly constituted English-speaking children from elementary grades. It has been noticed that there was a lack of variance in the certain aspect of morphological awareness (e.g., inflectional knowledge) among English-speaking children from more advanced grades because the foundational linguistic skill such as morphological awareness became well-developed as children grew up (Kuo and Anderson, 2006). It has also been found that morphological awareness can contribute to reading comprehension both directly and indirectly *via* a range of other foundational language skills such as lexical skill (Kieffer et al., 2013) and phonological skill (Nagy et al., 2006). However, less is known about how morphological awareness interacts with cognitive skills.

A major difference between Chinese and alphabetic languages is that in Chinese, there is a lack of correspondence between the orthographic units and phonemes as seen in alphabetic languages. The orthographic unit needs to be recognized as a whole before activating the phonology (Florit and Cain, 2011). As an analytic language, 75% of Chinese words contain two or three morphemes that rely heavily on morphological knowledge to access semantic clues and make inference about words’ meaning (Chen et al., 2009). For example, 上下车 (get on the car/get off the car) in Chinese is formed by three morphemes or characters, 上 (get on), 下 (get off), and 车(car). Readers can make inferences about word meaning based on the three morphemes. Therefore, morphological knowledge is essential for Chinese reading comprehension.

Most studies on the role of morphological awareness in Chinese reading focus on native Chinese speakers. It was found that morphological awareness directly contributed to reading comprehension and indirectly predicted reading comprehension through word reading for Chinese kindergarteners (Pan and Lin, 2023), Grade 1 to 2 Chinese pupils (Cheng et al., 2017), Grades 3 and 4 children (Qiao et al., 2021). However, Zhang's (2014) study on Chinese second graders found that the effect of morphological awareness on reading comprehension was mediated by lexical inference ability and vocabulary knowledge and that morphological awareness had no significant contribution to reading comprehension after controlling for the two mediating factors. It should be noticed that morphological awareness was measured differently in these studies. Zhang (2014) adopted a morpheme recognition and a morpheme discrimination task, while others employed either a compound production task (Cheng et al., 2017) or a morphological construction task (Qiao et al., 2021; Pan and Lin, 2023).

Only a small number of recent studies have examined morphological awareness in L2 Chinese reading comprehension. A recent study on L1 English learners of L2 Chinese showed that morphological awareness (as measured by morpheme segmentation and morpheme discrimination) directly contributed to reading comprehension, while lexical inference directly contributed to reading and mediated the effect of morphological awareness on reading comprehension (Zhang et al., 2021). Another study on Chinese as a heritage language adult learners found that morphological awareness (as measured by structural awareness and functional awareness) directly contributed to reading comprehension (in the form of lexical inference and passage comprehension) and mediated the effect of vocabulary knowledge on reading comprehension (Zhang and Koda, 2018).

These studies showed that although morphological awareness is essential in explaining Chinese reading performance, the route it takes to affect comprehension varies when different skills are added to the model. Until recently, few studies integrate a full range of cognitive skills such as monitoring and working memory into a model of Chinese reading. We now turn to see why these cognitive skills are essential in understanding Chinese reading comprehension.

#### 2.2.2. Basic cognitive skill: Working memory

Working memory, as the basic cognitive function, is related to a set of processes occurring during reading comprehension, such as inhibition, deployment of attention, information update, and so on. It has a significant impact on the development of reading comprehension capacity (Perfetti and Stafura, 2014). Working memory is more than a passive repository otherwise it would be reduced to a mere container of the incoming information. It is a cognitive system serving as a threshold that can control and manipulate the information going into the permanent memory. To be more precise, working memory is a cognitive system limited in capacity, which can affect higher-order cognitive activities such as reading comprehension, language production, inference making, and problems solving (Oberauer et al., 2003).

Working memory not only integrates information but also inhibits irrelevant information in order to release the limited processing capacity that can be used for drawing complex inferences. According to the Structure Building Framework (Gernsbacher, 1990), the goal of comprehension is the building of a cohesive mental representation, or structure, which relies on three processes, laying a foundation, mapping, and shifting. When there is an inconsistency in the information flow, working memory will suppress the irrelevant information and shift it to another subset. If there the information is consistent, working memory will be reinforced.

Working memory inhibitory mechanisms consist of two important skills: blocking information flow and deleting irrelevant information (Hasher and Zacks, 1988). These skills ensure that irrelevant information is deleted and unable to enter working memory so that only relevant information gets processed. Readers with stronger working memory can better suppress the information (Yeari and van den Broek, 2011). The stronger the suppression is, the more efficient working memory becomes, and the better reading comprehension is achieved. The inhibitory mechanism can promote working memory and contribute to the construction of a coherent mental representation (Perfetti and Stafura, 2014). Chinese reading comprehension consumes more cognitive resources than alphabetical reading (Diamond, 2013). Recent studies on Chinese reading have shown that working memory supports reading comprehension (Weng et al., 2016; Liu and Wu, 2022; Yeung, 2022). Readers with poor working memory are not likely to perform well in reading. However, it is still unclear how working memory would influence L2 Chinese reading in a hierarchical model of comprehension.

#### 2.2.3. Higher-order cognitive skills

Inference making and comprehension monitoring were the two higher-order cognitive skills investigated in this study. Inference making is crucial for proficient reading and pertains to higher-order cognitive skills (Singer et al., 1994). To construct mental representations of meaningful events in a text, readers need to establish relations and draw inferences based on the context and background (Currie and Cain, 2015). Readers make inferences about local and global coherence to fill in details not explicitly stated in the text (Graesser et al., 1994). As a result, inference making skill ensures that information in the text is integrated with information from other sentences and world knowledge, filling in the information gaps and promoting local and global coherence. Readers’ inference making skills predict their reading comprehension in both English (Cain et al., 2001) and Chinese (Zhao et al., 2022). However, less is known about the role that inference making plays in a hierarchical model of the linguistic-cognitive skills for L2 Chinese reading comprehension.

Comprehension monitoring skills will be employed during the unfolding of text and the integration of new information (Zhao et al., 2022). Readers with strong reading comprehension skills are more likely to identify contradictory information and monitor their comprehension more effectively and incorporate it into a situation model (Cain, 2009). As readers monitor their understanding of words and sentences, they evaluate their propositions and integrate their knowledge of word usage and word knowledge (Kinnunen et al., 1998). Readers with strong comprehension skills are more likely to engage in strategic processing, discover connections between text elements, and draw on prior knowledge to promote understanding. In contrast, readers with weaker comprehension skills are less likely to achieve consistent monitoring of their comprehension and often fail to suppress or repair inconsistencies within a text (Kinnunen et al., 1998). A study on English-speaking children has shown that comprehension monitoring is an important variable contributing to reading comprehension after controlling other aspects (decoding, vocabulary, and working memory) of language comprehension (Yeomans-Maldonado, 2017). Zhao et al. (2022) found direct relation of comprehension monitoring to Chinese reading comprehension. The case of L2 Chinese readers is less discussed.

#### 2.2.4. Higher-order skills as mediators

According to RSF theory, the higher-order cognitive skills (e.g., monitoring and inference making) might mediate the effects of the foundational language skills on reading comprehension. Empirical evidence has shown, for example, that the skills for generating different types of inferences are heavily dependent on different types of vocabulary knowledge (Oakhill et al., 2015). Another study on English-speaking children found that the effect of vocabulary knowledge on reading comprehension was mediated by inference making skill (Silva and Cain, 2015).

Similar to the results of inference making studies, there has been evidence showing that foundational language skills underpin the comprehension monitoring skill (Kim, 2015; Zargar et al., 2020). Zargar et al.’s (2020) eye-tracking study on English-speaking children found that the gaze duration (an indicator of comprehension monitoring in the form of comprehension evaluation) was dependent on vocabulary knowledge. Kim’s (2015) study on Korean-speaking children found that vocabulary and syntactic knowledge were directly related to listening comprehension and also indirectly predicted listening comprehension *via* comprehension monitoring. It also found that listening comprehension and word reading mediated the effects of all language skills and cognitive skills on reading comprehension. The importance of syntactic knowledge for comprehension monitoring lies in the fact that it allows readers to better understand the grammatical relations between words and sentences and detect syntactic errors.

### 2.3. Purpose of this study

Despite the fact that previous studies have found evidence that supports the significant contributions of basic cognitive skills, foundational language skills and higher-order cognitive skills to Chinese reading comprehension, the hierarchical relationships among these skills have not been examined in a single synthesized model. Several previous studies have started to construct such an integrated model of reading comprehension. For example, Kim’s (2017) model of native English children’s reading comprehension covered working memory, vocabulary, grammatical knowledge, inference, comprehension monitoring, word reading, and listening comprehension. Pan and Lin’s (2023) model of Chinese children’s reading comprehension covered nonverbal IQ, phonological awareness, morphological awareness, orthographic knowledge, vocabulary knowledge, word reading, and listening comprehension. These two models both drew on the two important factors in the simple view of reading, word reading and listening comprehension (Hoover and Gough, 1990). However, these two skills can be further divided into componential skills. Treating them as a whole may blur the hierarchical relationships among the componential language and cognitive skills in reading. Zhao et al.’s (2022) model shared a similar attempt with the current study. She explored the direct and indirect effects of language skills (vocabulary, syntactic knowledge, and orthographic knowledge) and cognitive skills (inference making and comprehension monitoring) on Chinese Grade 3 pupils’ reading comprehension. However, she did not include key factors such as morphological awareness and working memory. Also, previous studies are mainly interested in native speakers of English or Chinese, few studies provide insights into the L2 Chinese learners’ reading performance.

To extend our understanding of the significance of the basic cognitive skill (working memory), foundational language skills (syntactic knowledge, lexical knowledge, orthographic knowledge, and morphological awareness), and higher-order cognitive skills (inference making and comprehension monitoring) in explaining Chinese reading comprehension, these skills were examined in one model in the current study. More importantly, the research also examined the direct and indirect contributions of each variable to gain the hierarchical relations among language skills, basic cognitive skill, and higher-order cognitive skills with respect to reading comprehension in L2 Chinese learners. Specifically, the study sought to answer two research questions:

1.How do these three facets (the basic cognitive skill, foundational language skills, higher-order cognitive skills) make their contributions to the L2 Chinese reading comprehension?
2.What are the direct and/or indirect routes each skill took to affect L2 Chinese reading comprehension?

Regarding the first research question, we hypothesized that Chinese reading comprehension would be influenced by language and cognitive skills, and each cognitive and language skill had a unique contribution to Chinese reading comprehension. Multiple regressions were used to determine each variable’s contribution.

Regarding the second question, based on previous research, language skills were intertwined with cognitive skills in reading comprehension, for example, the association between syntactic knowledge and executive function (Hung, 2021), the relationship between vocabulary and inferencing (Oakhill et al., 2015; Silva and Cain, 2015), and the relationship between vocabulary and monitoring (Zargar et al., 2020). The higher-order cognitive skills may mediate the relationship between language skills and reading comprehension. Therefore, we used a path analysis method to determine how well our proposed model fits current data and examine the significance of each direct and indirect path in the model. We hypothesized that the basic cognitive skill and foundational language skills contribute to reading comprehension *via* higher-order cognitive skills which function as mediators.

## 3. Method

### 3.1. Participants

A total of 252 first-year college students ranging from 18 to 21 years old were enrolled from four different universities in China. One student dropped out, so 251 students took the tests (131 males, 120 females, *M*_age_ = 19.73 years, *SD* = 1.28). They were international students from Pakistan, Indonesia, Malaysia, and Laos. Their mother tongues belong to alphabetic languages different from Chinese. All of them passed the HSK2-level language exam before arriving in China.

All participants learned HSK3-level Chinese curriculum including reading as a core subject, not as a separate one. Chinese curriculums focus on the teaching and learning of texts from HSK3-level Standard Course. At the end of HSK3-level Chinese curriculum, participants have grasped 600 Chinese characters with basic proficiency in Chinese reading. According to their teachers, all participants exhibited no hearing impairments or language impairments. Participant permission was obtained for the study.

### 3.2. Measures

#### 3.2.1. Working memory test

Working memory tests are a battery of tasks that measures people’s capacity to temporarily store and process various types of information. These tasks can be categorized into simple/complex linguistic tasks and simple/complex non-linguistic tasks. Reading span tasks and operation span tasks are widely used in empirical studies. The present study adopted a linguistic working memory task. Adapted from Kim’s (2016) study, a reading span task was carried out on E-Prime 2.0. It contained 30 experimental trials and 5 practice trials. In each trial, participants saw a sentence of 7 to 10 Chinese characters followed by a Chinese word in bold type (e.g., 小王，请帮我开门，谢谢。**北京**. *Xiao Wang, open the door for me please. Beijing.*). All the trial sentences were selected from HSK3-level syllabuses. The participants were tested in two dimensions. The first dimension was the capacity to store linguistic information. After each sentence was presented, participants were required to identify whether a new word on the screen matched the last word of the trial sentence. The second dimension was comprehension. A statement about the trial sentence was presented and the participants were asked to judge whether the statement was correct or not (yes/no response). The task consisted of a total of 15 items. Each item is given 2 marks. In order to get 2 marks, participants had to recall new words on the screen and respond to yes/no questions. The maximum score for the task was 30. Cronbach’s alpha for this working memory test was α = 0.91.

#### 3.2.2. Chinese reading comprehension test

Reading comprehension capacity can be tested at sentence and discourse level (Tunmer and Chapman, 2012). This study administered a Chinese reading comprehension test adapted from the HSK3-level texts. There were 20 multiple choice questions for them to answer. Each item was given one mark. At sentence level, the participants were presented with 10 sentences, each with a blank. They were required to select appropriate answers from the choices provided. For example:


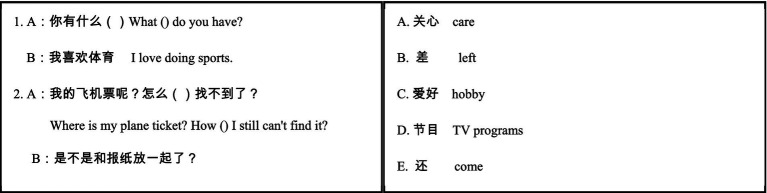


At discourse level, the participants were presented with 10 paragraphs, each with one or two questions. They were required to select appropriate answers from the choices provided. For example:


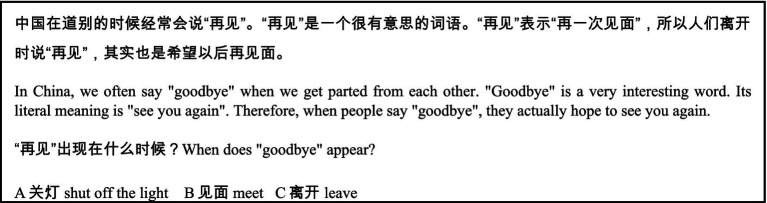


The maximum score for the task was 20. The reliability of the comprehension test was 0.81.

#### 3.2.3. Linguistic knowledge tests

The linguistic knowledge of participants was measured in terms of four aspects: syntactic knowledge, lexical knowledge, orthographic knowledge, and morphological awareness. Totally 60 test items (15 items for each type of knowledge and each item one mark) were selected or adapted from HSK3-level examinations and the HSK3-level syllabus. The maximum score for the task was 60.

Syntactic knowledge was assessed by using a cloze task (Cronbach’s alpha at 0.87). There were 15 items, and each item was given 1 mark. The total score was 15 marks. The highly frequent use of conjunctions is a prominent feature of Chinese syntax and the knowledge about conjunctions can be regarded as an indicator of syntactic knowledge (Li and Thompson, 1981). The participants were presented with sentences missing conjunction words and required to select appropriate answers.


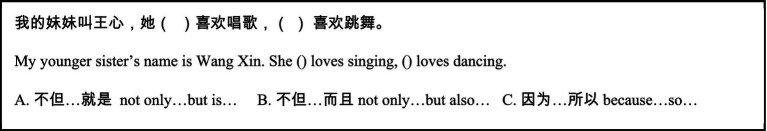


Lexical knowledge was measured by two tasks. The first task was to choose synonyms for underlined words in a sentence or paragraph. The second task was to choose appropriate words to complete a sentence or a paragraph. Altogether, there were 15 items in the two tasks, with 7 items for choosing synonyms and 8 items for choosing appropriate words and each item was given 1 mark. The total score was 15 marks. The reliability of this vocabulary test was 0.89.

Orthographic knowledge was assessed using a Chinese character decision task containing 5 real Chinese characters, 5 non-characters, and 5 pseudo-characters. The design of the non-characters and pseudo-characters followed a fundamental principle that possible constituents (radicals and strokes) of a Chinese character should be placed in their legal positions (Liu et al., 2017). There were 15 items, and each judgement was given 1 mark. The total score was 15 marks. The reliability of the orthographic knowledge test was 0.91.

The morphological awareness test was adapted from that of McBride-Chang et al. (2003). The morphological awareness in the study was composed of a test on homophones and a test on compounds. The homophone production test (Cronbach’s alpha at 0.83) is intended to measure participants’ knowledge of homophones. For example, participants were asked to produce a word (e.g., 时间 time) using a Chinese character时shí and then produce words (e.g., 石头stone、食堂canteen、实验experiment) using its homophones such as 石shí, 食shí, 实shí. Two solutions were required for each target character. Each correct solution was given a score of 0.5. The total number of test items was 10. The compounding semantic morpheme test (Cronbach’s alpha at 0.79) required participants to produce a compound word based on a specific description of a concept. An example question would be: “If a type of oil is made of sesame, then it is called sesame oil (芝麻油). If a type of oil is made of peanuts, what should we call it?” The answer should be peanuts oil (花生油). Similarly, each correct answer was given 1 mark. There is no point for participants who spelled incorrectly. There were 10 items. The total score was 15 marks.

#### 3.2.4. Higher-order cognitive skill tests

The inference making task and the comprehension monitoring task were developed to test whether the participants derive coherent information and detect inconsistencies in stories. Both tasks were administered on Eprime 2.0. The inference making task was adapted from Muijselaar (2018). It included 10 stories at the difficulty level appropriate for HSK3-level readers. Each story was followed by one question. After reading the stories, questions were presented on the screen. Participants were given one mark if they answer one question correctly. The total score was 10. In inference making test, the reliability of the inferencing was 0.83.

The monitoring task was adapted from Kim (2015). It still included 10 stories at the difficulty level appropriate for HSK3-level readers, with 7 information inconsistencies stories, and 3 normal stories. Each story was presented on the screen and followed by a true-or-false question. Each question was given 1 mark, and the total score was 10. The reliability of the monitoring task was 0.87.

### 3.3. Data analysis strategy

The data were analyzed using three major statistical approaches. Correlation analysis was conducted to investigate the relationship between the variables. Multiple regression methods were used to determine the unique contribution of each cognitive and language skill to reading comprehension. Correlation and hierarchical multiple regression were conducted by SPSS 28.0. Path analysis was conducted by Mplus 8.3 (Muthén and Muthén, 2017), which is used for determining how well our proposed model fit the available data, as well as evaluating the significance of each direct and indirect path.

## 4. Results

### 4.1. Descriptive analysis and preliminary analysis

The results of descriptive statistics and correlation analysis of the variables were shown in Table 1. Table 1 included the mean and *SD*, skewness, kurtosis as well as the bivariate correlations among the measures. Skewness and kurtosis of all variables are within an acceptable range, demonstrating normal distribution. Correlation analyses used the Pearson correlation test. L2 Chinese reading grade was weakly to strongly correlated with fundamental skills (orthographic knowledge, vocabulary, syntactic knowledge, morphological knowledge), basic cognitive skills (working memory) and high-order cognitive skills (inference making and monitoring) in the Pearson correlation test (0.16 ≤ *r*s ≤ 0.64, *p*s < 0.01). Higher-order cognitive skills (inference making and monitoring) were strongly related to L2 Chinese reading (0.63 ≤ *r*s ≤ 0.64); There was a weak to strong relationship between basic cognitive skills (working memory), fundamental skills (orthographic knowledge, vocabulary, syntactic knowledge, morphological knowledge) and high-order skills (inference making and monitoring) (0.21 ≤ rs ≤ 0.66).

**Table 1:** Descriptive statistics and bivariate correlations among variables.

	*M* (*SD*)	Orthographic knowledge	Vocabulary	Syntactic knowledge	Morphological knowledge	Working memory	Inference making	Monitoring	Chinese reading
Orthographic knowledge	10.64 (1.77)	1							
Vocabulary	9.85 (1.64)	0.36**	1						
Syntactic knowledge	10.31 (1.53)	0.24**	0.15*	1					
Morphological knowledge	10.03(2.21)	0.69**	0.42**	0.34**	1				
Working memory	25.41 (1.83)	0.54**	0.54*	0.48**	0.61**	1			
Inference making	5.62 (1.71)	0.45**	0.21**	0.18**	0.66**	0.38**	1		
Monitoring	5.39 (1.89)	0.46**	0.56**	0.50**	0.59**	0.53**	0.30**	1	
Chinese reading	15.84 (1.78)	0.16**	0.24**	0.26**	0.64**	0.66**	0.63**	0.64**	1
								
Skewness		0.65	−0.57	−0.79	−0.77	−1.7	0.41	−0.7	0.54
Kurtosis		0.54	0.08	0.19	0.21	0.94	0.55	0.23	0.32

*p < 0.05; **p < 0.01; ***p < 0.001.

### 4.2. Hierarchical multiple regression

A hierarchical multiple regression was performed to figure out the contributions of these variables to Chinese reading comprehension. Orthography, vocabulary, syntactic knowledge, morphology, working memory, inferencing and monitoring were independent variables; L2 Chinese reading was the dependent variable. In multiple regressions, the unstandardized coefficient *B*, standardized coefficient β, standard error (*SE*), ∆*R*^2^, and *R*^2^ are presented in Table 2. Standardized coefficient β was adopted to interpret the contributions of each variable (Jordan et al., 2013). All variables made statistically significant contributions to Chinese reading comprehension. Orthographic knowledge (β = 0.01, *p* < 0.05), lexical knowledge (β =0.05, *p* < 0.05), syntactic knowledge (β = 0.05, *p* < 0.01), morphological awareness (β = 0.40, *p* < 0.01), working memory (β = 0.22, *p* < 0.01), inference making (β = 0.23, *p* < 0.01), and comprehension monitoring (β = 0.21, *p* < 0.01) had a significant influence on Chinese reading comprehension. Overall, the basic cognitive skills, foundational language skills and higher-order cognitive skills explained 80% (*F* (7, 243) =72.89; *p* < 0.01) of the variances in L2 Chinese reading comprehension.

**Table 2:** Hierarchical multiple regression of the model predictors on reading comprehension.

Measures	L2 Chinese Reading Comprehension				
	*B*	*SE*	β	Δ*R*^2^	*R* ^2^
Orthography	0.01	0.03	0.01*	0.01	0.8
Vocabulary	0.06	0.03	0.05*	0.04	
Syntax	0.06	0.03	0.05**	0.05	
Morphology	0.33	0.04	0.40**	0.23	
Working memory	0.23	0.05	0.22**	0.17	
Inferencing	0.25	0.05	0.23**	0.16	
Monitoring	0.23	0.05	0.21**	0.14	

*p < 0.05; **p < 0.01.

Another purpose of the analysis was to determine the amount of unique variance in L2 Chinese reading that could be explained by each independent variable, respectively. In order to achieve the purpose, hierarchical multiple regressions were conducted. In the first step of each regression, all other variables were entered except for one, which was entered in the second step to determine the unique variance explained by that particular variable. Statistical analysis revealed that each of these variables contributed to the L2 Chinese reading after accounting for all other variables: orthographic knowledge 1% (*p* < 0.01), vocabulary 4% (*p* < 0.01), syntactic knowledge 5% (*p* < 0.01), morphological knowledge 23% (*p* < 0.01), working memory 17% (*p* < 0.01), inference making 16% (*p* < 0.01) and comprehension monitoring 14% (*p* < 0.01). According to Cohen (1988) *f*^2^’s definition of effect sizes, small effect sizes as 0.02, medium effect sizes as 0.15, and large effect sizes as 0.35. The effect sizes of morphological skills (Cohen *f*^2^ = 0.85), working memory (Cohen *f*^2^ = 0.55), inference making (Cohen *f*^2^ = 0.5) and monitoring (Cohen *f*^2^ = 0.4) are large. The effect sizes of vocabulary (Cohen *f*^2^ = 0.2) and syntactic knowledge (Cohen *f*^2^ = 0.25) are medium. The effect size of the orthography skill (Cohen *f*^2^ = 0.02) was small.

### 4.3. A path analysis

The path analysis was carried out using Mplus 8.3 (Muthén and Muthén, 2017) and the full information maximum likelihood estimator. Model fits were evaluated by the following indices: the χ2 statistic, comparative fit index (CFI), Tucker-Lewis index (TLI), root mean square error of approximation (RMSEA), and Standardized Root Mean Square Residual (SRMR). It was suggested that chi-square test should be used to compare model fits and the model was rejected if *p* < 0.05 (Hu and Bentler, 1999). A good fit of the model is indicated by the *χ*^2^ statistic to degrees of freedom ratio (*χ*^2^/*df*) equal to or less than 3 (Mclver and Carmines, 1981), TLI and CFI equal to or greater than 0.95 (Kline, 2005). A cutoff value close to 0.08 for SRMR and 0.06 for RMSEA are needed to report a good fit (Hu and Bentler, 1999). RMSEA values below 0.08 and SRMR equal to or less than 0.05 indicate an excellent model fit (Kline, 2005). The model had a good fit shown in Table 3.

**Table 3:** A path analysis model fit.

Model fit indices	*χ* ^2^	*Df*	*P*	CFI	TLI	RMSEA	SRMR
	6.818	6	0.338	0.998	0.995	0.023	0.026

**Figure 1:**
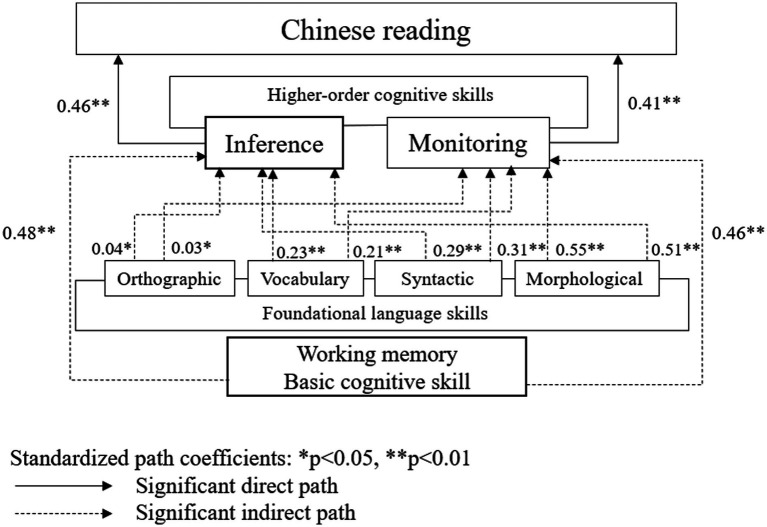
Standardized structural regression weights for the direct and indirect effects of the predictors in the L2 Chinese reading model.

All the statistically significant paths and their standardized path coefficients are reported in the model, as shown in Figure 1 and Table 4. First, we focused on the direct relationship between three facets and L2 Chinese reading comprehension. The path coefficients (𝛽) in the structural model ranging from 0 to 0.1, 0.11 to 0.30, 0.31 to 0.50, and > 0.50 are indicative of weak, modest, moderate, and strong effect sizes (Hair and Alamer, 2022). Statistically significant evidence was found for the direct effect of cognitive skills, inference making (β = 0.46, *p* < 0.01, moderate effect sizes) and comprehension monitoring (β = 0.41, *p* < 0.01, moderate effect sizes), on L2 Chinese reading. Inference making was predicted by orthographic knowledge (β = 0.04, p < 0.05, modest effect sizes), vocabulary (β = 0.23, *p* < 0.01, modest effect sizes), syntactic knowledge (β = 0.29, *p* < 0.01, modest effect sizes), morphological knowledge (β = 0.51, *p* < 0.01, strong effect sizes) and working memory (β = 0.48, *p* < 0.01, moderate effect sizes). Monitoring was predicted by orthographic knowledge (β = 0.03, *p* < 0.05, modest effect sizes), vocabulary (β = 0.21, *p* < 0.01, modest effect sizes), syntactic knowledge (β = 0.31, *p* < 0.01, moderate effect sizes), morphological knowledge (β = 0.55, p < 0.01, strong effect sizes) and working memory (β = 0.46, p < 0.01, moderate effect sizes).

**Table 4:** Direct, indirect, and total effect estimates of the model predictors on L2 Chinese reading.

	Predictors	Total effect	Direct effect	Indirect effect
Chinese reading	Orthographic knowledge	0.03	—	0.03
	Vocabulary	0.19	—	0.19
	Syntactic knowledge	0.26	—	0.26
	Morphological awareness	0.46	—	0.46
	Working memory	0.41	—	0.41
	Inference making	0.46	0.46	—
	Comprehension monitoring	0.41	0.41	—

Second, we focused on the indirect relationship between three facets and L2 Chinese reading comprehension. Orthographic knowledge, vocabulary, syntactic knowledge, morphological knowledge and working memory had indirect effects on Chinese reading *via* two routes, inference making or comprehension monitoring. Inference making was predicted by Orthographic knowledge (ab = 0.018, 95% CI [0.012, 0.093]; weak effect sizes), vocabulary (ab = 0.106, 95% CI [0.019, 0.103]; modest effect sizes), syntactic knowledge (ab = 0.133, 95% CI [0.023, 0.119]; modest effect sizes), morphological knowledge (ab = 0.235, 95% CI [0.105, 0.218]; modest effect sizes) and working memory (ab = 0.221, 95% CI [0.093, 0.220]; modest effect sizes). Comprehension monitoring was predicted by orthographic knowledge (ab = 0.073, 95% CI [0.014, 0.099]; weak effect sizes), vocabulary (ab = 0.086, 95% CI [0.021, 0.112]; weak effect sizes), syntactic knowledge (ab = 0.127, 95% CI [0.020, 0.116]; modest effect sizes), morphological knowledge (ab = 0.226, 95% CI [0.102, 0.211]; modest effect sizes) and working memory (ab = 0.189, 95% CI [0.087, 0.191]; modest effect sizes). In general, the effect sizes of orthographic knowledge and vocabulary in L2 Chinese reading are modest, even though they are statistically significant. Therefore, the results of the path analysis highlight the indirect effects of working memory, morphological knowledge and syntactic knowledge on L2 Chinese reading comprehension *via* inference making and comprehension monitoring.

## 5. Discussion

For the first question, how do these three facets (the basic cognitive, foundational language skills, and higher-order cognitive skills) make contributions to L2 Chinese reading comprehension? This study used hierarchical multiple regressions to examine the unique contributions of the three facets to L2 reading in Chinese. The results showed that altogether these factors explained 80% of the variances in L2 Chinese reading. The results are partly supported by evidence from Zhao et al. (2022) model. We extended prior research findings by (a) including morphological awareness and working memory in the multiple regressions model; (b) using data from L2 Chinese learners.

The present model explained substantial variances (80%) in L2 Chinese reading comprehension. Morphological knowledge explained the largest variance, that is 23% of the variance in Chinese reading comprehension, compared with other linguistic skills (i.e., vocabulary 4%, syntactic knowledge 5%, orthographic knowledge 1%). The importance of morphological awareness was found in L1 English children (Carlisle, 2000; Nagy et al., 2006; Tong et al., 2011; Kieffer et al., 2013), L1 Chinese children (Cheng et al., 2017; Qiao et al., 2021; Pan and Lin, 2023), heritage learners of Chinese (Zhang and Koda, 2018). There is a lack of correspondence between the orthographic units and phonemes in Chinese, which is different from alphabetic languages. This requires native Chinese speakers to rely on morphological knowledge, especially L1 Chinese children (e.g., Cheng et al., 2017). They need to develop a sensibility of how Chinese words represent meaning at the morphemic level because Chinese has a logography that contains a large number of homophones and polysemes. These structures are challenging for L1 Chinese learners and can interfere with comprehension (Leong and Ho, 2008). L1 Chinese readers with stronger morphological skills have quicker access to the lexicon and are able to extract meaning more easily from the text. Especially when they read words they do not already know, a morphological analysis may assist them in making an educated guess about the semantic and syntactic category of an unknown word. In the current study, the importance of morphological awareness was extended to L2 Chinese learners. The participants were college-level international students from alphabetic language backgrounds with well-developed cognitive skills and ample reading experience in their native language, but the results showed that they still relied on morphological knowledge. However, it should be noticed that morphological awareness influenced L2 Chinese reading indirectly through cognitive skills, which is an interesting finding we will now turn to.

For the second question, what are the direct or indirect roles each facet plays in the process of L2 Chinese reading comprehension?

The results indicated that that working memory was directly related to inference making and monitoring and indirectly contributed to L2 Chinese reading comprehension *via* the two higher-order cognitive skills. These cognitive skills are cooperating with each other when supporting reading comprehension. Working memory holds and retrieves relevant information in readers’ mind when processing incoming language input. At the same time, working memory inhibits guessing errors when readers encounter contradictory information (Van Reybroeck and De Rom, 2020). For example, when Chinese readers encounter an orthographic neighbor, which replaced strokes with an orthographically close Chinese character (such as 友 (friends) v.s. 发(hair)), poor readers may fail to monitor and inhibit Chinese lexical orthographic neighbors in lexical competition, consequently causing a negative influence on the Chinese reading comprehension. An inference in reading comprehension is the process of filling in information gaps by manipulating the clues stored in working memory. According to Yuill and Oakhill (1991), a lack of adequate inferencing occurs when one is not aware of the fact that inferencing is necessary, when one lacks the background knowledge, or when one’s working memory is inadequate.

This finding extended the existing research findings about the role of working memory in Chinese reading (Weng et al., 2016; Liu and Wu, 2022; Yeung, 2022), showing a more complex relationships among working memory, inference making, comprehension monitoring and L2 Chinese reading comprehension.

A notable result in the current study was that inferencing and monitoring mediated the relationship between morphological awareness and reading comprehension. This is in line with the findings by Ke and Koda (2017) who have observed that L1 English learners of Chinese who were more sensitive to the morphological structure of multi-character words were more successful in inferring the meanings of unfamiliar words.

For native speakers of alphabetic language such as English, it has been found that morphological awareness can contribute to reading comprehension directly or indirectly *via* a range of other foundational language skills such as vocabulary (Kieffer et al., 2013) and phonological awareness (Nagy et al., 2006). In our study, morphological awareness and other language skills turned out to be parallel factors whose effects on reading comprehension was mediated by higher-order cognitive skills. Previous studies (e.g., Nagy et al., 2006; Kieffer et al., 2013) did not consider the hierarchal relationships among cognitive skills and language skills. The variations among these findings could be explained by the specific component skills under investigation in different models.

Our findings are also slightly different from a recent study on L1 English learners of L2 Chinese (Zhang et al., 2021). They found that morphological awareness directly contributed to reading comprehension, while lexical inference directly contributed to reading and mediated the effect of morphological awareness on reading comprehension. In our case, we only found indirect contribution of morphological awareness to L2 Chinese reading through the two higher-order cognitive skills (i.e., inference making and monitoring). The discrepancy may be explained by different measurements of morphological awareness. The morphological awareness in the current study was composed of a test on homophones and a test on compounds, while Zhang et al. (2021) adopted a morpheme segmentation and a morpheme discrimination task. In addition, Zhang et al. (2021) only include a narrow range of reading-related skills (morphological awareness, vocabulary, and lexical inference), which may not be able to unveil a full picture, as shown in this study, of the hierarchal relationships among varying language and cognitive skills in reading comprehension.

In addition to morphological knowledge, our findings showed that inference making and monitoring skills mediated the relationship between syntactic knowledge and L2 Chinese reading comprehension. Chinese differs from alphabetic languages in several ways in terms of the syntactic features such as the absence of an inflectional system, more flexible word order, and more extensive use of connectives (Li and Thompson, 1981). The mediating role of higher-order cognitive skills may be explained by the possibility that L2 Chinese readers need to consume more cognitive resources during inference making and monitoring to comprehend Chinese sentences. Hung’s (2021) recent study also found the intertwined relationship between syntactic skills and cognitive skills and found an indirect effect of executive function (as measured by working memory tasks and inhibition tasks) on Chinese Grade 2 and 3 students’ reading comprehension through syntactic awareness (as measured by a word order correction task). It should be noticed that executive function covered some aspects of working memory and monitoring. In Hung’s study, indirect effects of executive function on reading through inference-making was not significant, refuting her original hypothesis that executive function can support inference making to achieve passage-level comprehension. However, in our model, working memory, inference making, and monitoring were measured independently and entered the model as three variables. We found that higher-order cognitive skills (i.e., inference making and monitoring) completely mediated the effect of the basic cognitive skill (i.e., working memory) on reading comprehension. Syntactic skill made no direct contribution to reading comprehension, but it made indirect contributions through both inference making and monitoring, which is in line with Zhao et al.’s (2022) findings from L1 Chinese readers. By adding more micro-level componential skills into a more holistic model of reading comprehension, we extended Hung’s findings and showed a clear hierarchical relationship between higher-order cognitive skills and the basic cognitive skill as well as the hierarchical relationship between higher-order cognitive skills and foundational language skills in reading comprehension.

Regarding inference making and monitoring, we found that monitoring and inference making made direct and moderate contributions to L2 Chinese reading comprehension, supporting the theory that developing a text representation with coherence requires evaluation and integration of initial propositions (Perfetti et al., 2005). We extend previous findings to a different population with L2 Chinese readers, demonstrating that higher-order cognitive skills are essential for both L2 Chinese readers, L1 Chinese readers (Zhao et al., 2022), and L1 English readers (Cain et al., 2004).

## 6. Limitation and future research

This study is limited in the following aspects. First, we did not include other factors that have been proven to be crucial to reading comprehension such as phonological awareness (Veenendaal et al., 2016; Lin and Zhang, 2021) and reading strategies (Yapp et al., 2021). Future research can explore more factors to establish a more comprehensive model of L2 Chinese reading. Second, the participants in this study have heterogeneous language backgrounds. Although their mother tongues belong to the broad alphabetic language family, the heterogeneity of their first language has not been controlled in the current study. First language may exert great influence on reading comprehension. For example, coherence relations have different forms across languages and first language may entail misuse of cohesive devices in another language (Wetzel et al., 2022). Future studies can take language background as a key variable in developing a more refined model of Chinese reading.

## 7. Conclusion

Reading is a complex process involving complex interactions between linguistic and cognitive skills. The results of this study showed that cognitive skills and foundational language skills can influence Chinese reading through direct and indirect pathways. In addition, there is a clear hierarchical relationship between the contributions of cognitive skills and linguistic skills. Higher-order cognitive skills completely mediated the contributions of foundational language skills and the basic cognitive skill. Also, a systematic understanding of reading comprehension needs to consider both general language-related issues and special features of a specific language. Chinese morphosyllabic orthography is different from many alphabetic languages and entails a large body of homophones. This uniqueness makes morphological awareness exceptionally important in Chinese reading comprehension. Future studies can further explore the role of other components of the linguistic-cognitive skills such as the central executive and the phonological loop of working memory in Chinese reading comprehension.

## Data availability statement

The raw data supporting the conclusions of this article will be made available by the authors, without undue reservation.

## Ethics statement

The studies involving human participants were reviewed and approved by Xi’an Jiao Tong University. The patients/participants provided their written informed consent to participate in this study.

## Author contributions

LY was responsible for conducting the research, writing and revising the manuscript. YX was responsible for writing and revising the manuscript. LY and YX responded to all the reviewers’ comments. QC helped in editing the final version of the manuscript. First authorship goes to LY. Second authorship goes to YX. Third authorship goes to QC. All authors contributed to the article and approved the submitted version.

## Funding

This work was supported by the Ministry of Education, People’s Republic of China (grant no. 18YJA740063).

## Conflict of interest

The authors declare that the research was conducted in the absence of any commercial or financial relationships that could be construed as a potential conflict of interest.

## Publisher’s note

All claims expressed in this article are solely those of the authors and do not necessarily represent those of their affiliated organizations, or those of the publisher, the editors and the reviewers. Any product that may be evaluated in this article, or claim that may be made by its manufacturer, is not guaranteed or endorsed by the publisher.
